# 2,2′-{1,1′-[Butane-1,4-diyl­bis(oxy­nitrilo)]di­ethylidyne}di-1-naphthol

**DOI:** 10.1107/S160053680901647X

**Published:** 2009-05-14

**Authors:** Wen-Kui Dong, Jian-Chao Wu, Yin-Xia Sun, Jian Yao, Jun-Feng Tong

**Affiliations:** a School of Chemical and Biological Engineering, Lanzhou Jiaotong University, Lanzhou 730070, People’s Republic of China

## Abstract

The title compound, C_28_H_28_N_2_O_4_, was synthesized by the reaction of 2-acetyl-1-naphthol with 1,4-bis­(amino­oxy)butane in ethanol. The molecule, which lies about an inversion centre, adopts a linear structure, in which the oxime groups and naphthalene ring systems assume an *anti* conformation. The intra­molecular inter­planar distance between parallel naphthalene rings is 1.054 (3) Å. Intra­molecular O—H⋯N hydrogen bonds are formed between the oxime nitro­gen and hydr­oxy groups.

## Related literature

For salen-type compounds, see: Atwood & Harvey (2001 ▶); Okabe & Oya (2000 ▶). For related structures, see: Dong *et al.* (2007 ▶, 2008 ▶).
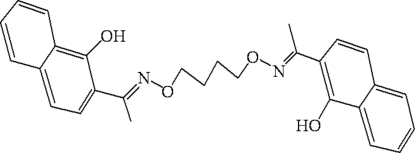


## Experimental

### 

#### Crystal data

C_28_H_28_N_2_O_4_
*M* *_r_* = 456.52
Triclinic, 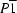

*a* = 6.9590 (15) Å
*b* = 8.6598 (18) Å
*c* = 10.596 (2) Å
α = 105.841 (2)°
β = 105.940 (2)°
γ = 91.689 (1)°
*V* = 586.9 (2) Å^3^
*Z* = 1
Mo *K*α radiation
μ = 0.09 mm^−1^
*T* = 298 K
0.50 × 0.43 × 0.20 mm

#### Data collection

Bruker SMART 1000 CCD area-detector diffractometer
Absorption correction: multi-scan (*SADABS*; Sheldrick, 1996 ▶) *T*_min_ = 0.958, *T*_max_ = 0.983
3020 measured reflections
2026 independent reflections
1350 reflections with *I* > 2σ(*I*)
*R*_int_ = 0.025

#### Refinement

*R*[*F* ^2^ > 2σ(*F* ^2^)] = 0.054
*wR*(*F* ^2^) = 0.173
*S* = 1.02
2026 reflections
154 parameters
H-atom parameters constrained
Δρ_max_ = 0.23 e Å^−3^
Δρ_min_ = −0.25 e Å^−3^

### 

Data collection: *SMART* (Siemens, 1996 ▶); cell refinement: *SAINT* (Siemens, 1996 ▶); data reduction: *SAINT*; program(s) used to solve structure: *SHELXS97* (Sheldrick, 2008 ▶); program(s) used to refine structure: *SHELXL97* (Sheldrick, 2008 ▶); molecular graphics: *SHELXTL* (Sheldrick, 2008 ▶); software used to prepare material for publication: *SHELXTL*.

## Supplementary Material

Crystal structure: contains datablocks global, I. DOI: 10.1107/S160053680901647X/hg2505sup1.cif

Structure factors: contains datablocks I. DOI: 10.1107/S160053680901647X/hg2505Isup2.hkl

Additional supplementary materials:  crystallographic information; 3D view; checkCIF report

## Figures and Tables

**Table 1:** Hydrogen-bond geometry (Å, °)

*D*—H⋯*A*	*D*—H	H⋯*A*	*D*⋯*A*	*D*—H⋯*A*
O2—H2⋯N1	0.82	1.84	2.557 (2)	145

**Fig. 1.**
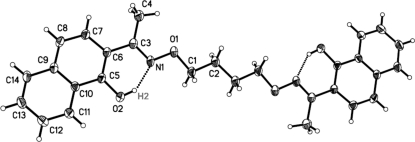
The molecule structure of the title compound with atom numbering [Symmetry codes: -x + 1, -y + 1, -z]. Displacement ellipsoids for non-hydrogen atoms are drawn at the 30% probability level.

## supplementary crystallographic information

### Comment

Salen-type compounds and their analogues are one of the most prevalent multidentate ligands in the field of modern coordination chemistry (Atwood & Harvey, 2001), which can coordinate to transition or rare earth ions yielding complexes with interesting properties that are useful in materials science and in biological systems (Okabe & Oya, 2000).

Herein, we report on the crystal structure of 2,2'-[(butane-1,4-diyldioxy)bis(nitriloethylidyne)]dinaphthol, shown in Fig. 1. The structure of the title compound consists of discrete C_28_H_28_N_2_O_4_ molecule, in which all bond lengths and angles are in normal ranges. The molecule is disposed about a crystallographic centre of symmetry with the (–CH═N—O-(CH)_4_—O—N═CH–) bridge adopting a linear-shaped structure. The oxime groups and naphthalene rings assume an anti conformation. This structure is not similar to what was observed in our previously reported series salen-type compounds containing four-methene bridge, which often adopt an E configuration (Dong *et al.*, 2007, 2008).

The two naphthalene rings in each molecule of the title compound are parallel and the distance between them is 1.054 (3) Å. Two intramolecular O—H···N hydrogen bonds are formed between the oxime nitrogen and hydroxy groups.

### Experimental

2,2'-[(Butane-1,4-diyldioxy)bis(nitriloethylidyne)]dinaphthol was synthesized according to our previous work (Dong *et al.*, 2007). To an ethanol solution (5 ml) of 2-acetyl-1-naphthol (760.0 mg, 2.02 mmol) was added dropwise an ethanol solution (3 ml) of 1,4-bis(aminooxy)butane (243.0 mg, 1.01 mmol). The mixture solution was stirred at 328–333 K for 75 h. After cooling to room temperature, the precipitate was filtered off, and washed successively three times with ethanol. The product was dried *in vacuo* and purified by recrystallization from ethanol to yield 618.6 mg (yield 67.0%) of powder. Single crystals were obtained by slow evaporation from a solution of ethanol/dichloromethane (1:2) of 2,2'-[(butane-1,4-diyldioxy)bis(nitriloethylidyne)]dinaphthol at room temperature for several weeks. Anal. Calcd. for C_28_H_28_N_2_O_4_: C, 73.66; H, 6.18; N, 6.14; Found: C, 73.61; H, 6.23; N, 6.09%.

### Refinement

Non-H atoms were refined anisotropically. H atoms were treated as riding atoms with distances C—H = 0.97 (CH_2_), C—H = 0.96 (CH_3_), 0.93 Å (CH), 0.82 Å (OH), and *U*_iso_(H) = 1.2 *U*_eq_(C) and 1.5 *U*_eq_(O).

### Crystal data

**Table:**

C_28_H_28_N_2_O_4_	*Z* = 1
*M**_r_* = 456.52	*F*(000) = 242
Triclinic, *P*1	*D*_x_ = 1.292 Mg m^−^^3^
Hall symbol: -P 1	Mo *K*α radiation, λ = 0.71073 Å
*a* = 6.9590 (15) Å	Cell parameters from 1246 reflections
*b* = 8.6598 (18) Å	θ = 2.5–27.6°
*c* = 10.596 (2) Å	µ = 0.09 mm^−^^1^
α = 105.841 (2)°	*T* = 298 K
β = 105.940 (2)°	Block, pale-yellow
γ = 91.689 (1)°	0.50 × 0.43 × 0.20 mm
*V* = 586.9 (2) Å^3^	

### Data collection

**Table:**

Bruker SMART 1000 CCD area-detector diffractometer	2026 independent reflections
Radiation source: fine-focus sealed tube	1350 reflections with *I* > 2σ(*I*)
graphite	*R*_int_ = 0.025
φ and ω scans	θ_max_ = 25.0°, θ_min_ = 2.1°
Absorption correction: multi-scan (*SADABS*; Sheldrick, 1996)	*h* = −8→8
*T*_min_ = 0.958, *T*_max_ = 0.983	*k* = −9→10
3020 measured reflections	*l* = −12→6

### Refinement

**Table:**

Refinement on *F*^2^	Primary atom site location: structure-invariant direct methods
Least-squares matrix: full	Secondary atom site location: difference Fourier map
*R*[*F*^2^ > 2σ(*F*^2^)] = 0.054	Hydrogen site location: inferred from neighbouring sites
*wR*(*F*^2^) = 0.173	H-atom parameters constrained
*S* = 1.02	*w* = 1/[σ^2^(*F*_o_^2^) + (0.096*P*)^2^ + 0.0813*P*] where *P* = (*F*_o_^2^ + 2*F*_c_^2^)/3
2026 reflections	(Δ/σ)_max_ < 0.001
154 parameters	Δρ_max_ = 0.23 e Å^−^^3^
0 restraints	Δρ_min_ = −0.24 e Å^−^^3^

### Special details

**Table:**

Geometry. All e.s.d.'s (except the e.s.d. in the dihedral angle between two l.s. planes) are estimated using the full covariance matrix. The cell e.s.d.'s are taken into account individually in the estimation of e.s.d.'s in distances, angles and torsion angles; correlations between e.s.d.'s in cell parameters are only used when they are defined by crystal symmetry. An approximate (isotropic) treatment of cell e.s.d.'s is used for estimating e.s.d.'s involving l.s. planes.
Refinement. Refinement of *F*^2^ against ALL reflections. The weighted *R*-factor *wR* and goodness of fit *S* are based on *F*^2^, conventional *R*-factors *R* are based on *F*, with *F* set to zero for negative *F*^2^. The threshold expression of *F*^2^ > σ(*F*^2^) is used only for calculating *R*-factors(gt) *etc*. and is not relevant to the choice of reflections for refinement. *R*-factors based on *F*^2^ are statistically about twice as large as those based on *F*, and *R*- factors based on ALL data will be even larger.

### Fractional atomic coordinates and isotropic or equivalent isotropic displacement parameters (Å^2^)

**Table:**

	*x*	*y*	*z*	*U*_iso_*/*U*_eq_	
N1	0.4299 (3)	0.3289 (2)	0.31934 (16)	0.0435 (5)	
O1	0.3505 (2)	0.37340 (18)	0.19981 (14)	0.0515 (5)	
O2	0.7182 (2)	0.30688 (18)	0.52155 (15)	0.0505 (5)	
H2	0.6665	0.3326	0.4519	0.076*	
C1	0.5112 (3)	0.4251 (3)	0.1562 (2)	0.0455 (6)	
H1A	0.5966	0.3396	0.1410	0.055*	
H1B	0.5923	0.5189	0.2259	0.055*	
C2	0.4211 (3)	0.4667 (3)	0.0252 (2)	0.0449 (6)	
H2A	0.3277	0.5459	0.0400	0.054*	
H2B	0.3460	0.3705	−0.0449	0.054*	
C3	0.2958 (3)	0.2877 (2)	0.3702 (2)	0.0402 (5)	
C4	0.0759 (3)	0.2939 (3)	0.3091 (2)	0.0627 (7)	
H4A	0.0578	0.3501	0.2408	0.094*	
H4B	0.0198	0.3496	0.3798	0.094*	
H4C	0.0091	0.1859	0.2673	0.094*	
C5	0.5732 (3)	0.2467 (2)	0.5630 (2)	0.0372 (5)	
C6	0.3690 (3)	0.2344 (2)	0.49419 (19)	0.0365 (5)	
C7	0.2306 (3)	0.1639 (2)	0.5453 (2)	0.0433 (5)	
H7	0.0937	0.1554	0.5009	0.052*	
C8	0.2912 (3)	0.1088 (2)	0.6565 (2)	0.0438 (6)	
H8	0.1961	0.0608	0.6851	0.053*	
C9	0.4976 (3)	0.1238 (2)	0.7294 (2)	0.0386 (5)	
C10	0.6397 (3)	0.1961 (2)	0.6838 (2)	0.0370 (5)	
C11	0.8449 (3)	0.2185 (3)	0.7606 (2)	0.0506 (6)	
H11	0.9400	0.2668	0.7321	0.061*	
C12	0.9047 (4)	0.1696 (3)	0.8761 (3)	0.0600 (7)	
H12	1.0397	0.1872	0.9270	0.072*	
C13	0.7640 (4)	0.0933 (3)	0.9182 (2)	0.0564 (7)	
H13	0.8068	0.0574	0.9955	0.068*	
C14	0.5661 (3)	0.0711 (3)	0.8475 (2)	0.0479 (6)	
H14	0.4743	0.0207	0.8770	0.057*	

### Atomic displacement parameters (Å^2^)

**Table:**

	*U*^11^	*U*^22^	*U*^33^	*U*^12^	*U*^13^	*U*^23^
N1	0.0575 (12)	0.0489 (11)	0.0329 (9)	0.0084 (9)	0.0176 (8)	0.0215 (8)
O1	0.0585 (10)	0.0695 (11)	0.0386 (8)	0.0082 (8)	0.0167 (7)	0.0329 (7)
O2	0.0468 (9)	0.0664 (10)	0.0556 (10)	0.0120 (7)	0.0240 (7)	0.0362 (8)
C1	0.0570 (13)	0.0519 (13)	0.0402 (12)	0.0112 (10)	0.0242 (10)	0.0234 (10)
C2	0.0573 (14)	0.0482 (12)	0.0360 (12)	0.0094 (11)	0.0180 (10)	0.0188 (9)
C3	0.0498 (12)	0.0394 (11)	0.0345 (11)	0.0036 (9)	0.0159 (9)	0.0121 (9)
C4	0.0544 (15)	0.0897 (19)	0.0525 (14)	0.0030 (13)	0.0111 (11)	0.0397 (13)
C5	0.0447 (12)	0.0363 (11)	0.0393 (11)	0.0080 (9)	0.0219 (9)	0.0152 (9)
C6	0.0443 (12)	0.0380 (11)	0.0313 (10)	0.0048 (9)	0.0155 (9)	0.0124 (8)
C7	0.0434 (12)	0.0523 (13)	0.0374 (11)	0.0003 (10)	0.0131 (9)	0.0178 (10)
C8	0.0487 (13)	0.0495 (13)	0.0404 (12)	−0.0015 (10)	0.0203 (10)	0.0182 (10)
C9	0.0505 (13)	0.0354 (11)	0.0375 (11)	0.0105 (9)	0.0202 (9)	0.0149 (9)
C10	0.0438 (12)	0.0367 (11)	0.0379 (11)	0.0138 (9)	0.0177 (9)	0.0157 (9)
C11	0.0466 (13)	0.0589 (14)	0.0597 (14)	0.0164 (11)	0.0218 (11)	0.0315 (11)
C12	0.0498 (14)	0.0742 (17)	0.0654 (16)	0.0220 (12)	0.0132 (12)	0.0376 (13)
C13	0.0643 (16)	0.0638 (15)	0.0534 (14)	0.0232 (12)	0.0170 (12)	0.0356 (12)
C14	0.0621 (15)	0.0493 (13)	0.0455 (13)	0.0138 (11)	0.0239 (11)	0.0262 (10)

### Geometric parameters (Å, °)

**Table:**

N1—C3	1.285 (3)	C5—C10	1.427 (3)
N1—O1	1.398 (2)	C6—C7	1.423 (3)
O1—C1	1.425 (2)	C7—C8	1.357 (3)
O2—C5	1.350 (2)	C7—H7	0.9300
O2—H2	0.8200	C8—C9	1.415 (3)
C1—C2	1.503 (3)	C8—H8	0.9300
C1—H1A	0.9700	C9—C10	1.413 (3)
C1—H1B	0.9700	C9—C14	1.414 (3)
C2—C2^i^	1.509 (4)	C10—C11	1.414 (3)
C2—H2A	0.9700	C11—C12	1.365 (3)
C2—H2B	0.9700	C11—H11	0.9300
C3—C6	1.475 (3)	C12—C13	1.400 (3)
C3—C4	1.498 (3)	C12—H12	0.9300
C4—H4A	0.9600	C13—C14	1.354 (3)
C4—H4B	0.9600	C13—H13	0.9300
C4—H4C	0.9600	C14—H14	0.9300
C5—C6	1.394 (3)		
			
C3—N1—O1	113.72 (17)	C5—C6—C7	117.71 (18)
N1—O1—C1	109.26 (15)	C5—C6—C3	122.01 (18)
C5—O2—H2	109.5	C7—C6—C3	120.26 (18)
O1—C1—C2	107.96 (18)	C8—C7—C6	122.4 (2)
O1—C1—H1A	110.1	C8—C7—H7	118.8
C2—C1—H1A	110.1	C6—C7—H7	118.8
O1—C1—H1B	110.1	C7—C8—C9	120.59 (19)
C2—C1—H1B	110.1	C7—C8—H8	119.7
H1A—C1—H1B	108.4	C9—C8—H8	119.7
C1—C2—C2^i^	112.2 (2)	C10—C9—C14	118.88 (19)
C1—C2—H2A	109.2	C10—C9—C8	118.87 (17)
C2^i^—C2—H2A	109.2	C14—C9—C8	122.24 (19)
C1—C2—H2B	109.2	C9—C10—C11	118.88 (18)
C2^i^—C2—H2B	109.2	C9—C10—C5	119.50 (18)
H2A—C2—H2B	107.9	C11—C10—C5	121.61 (19)
N1—C3—C6	116.56 (18)	C12—C11—C10	120.5 (2)
N1—C3—C4	122.53 (18)	C12—C11—H11	119.8
C6—C3—C4	120.91 (18)	C10—C11—H11	119.8
C3—C4—H4A	109.5	C11—C12—C13	120.4 (2)
C3—C4—H4B	109.5	C11—C12—H12	119.8
H4A—C4—H4B	109.5	C13—C12—H12	119.8
C3—C4—H4C	109.5	C14—C13—C12	120.6 (2)
H4A—C4—H4C	109.5	C14—C13—H13	119.7
H4B—C4—H4C	109.5	C12—C13—H13	119.7
O2—C5—C6	122.92 (17)	C13—C14—C9	120.8 (2)
O2—C5—C10	116.22 (18)	C13—C14—H14	119.6
C6—C5—C10	120.86 (18)	C9—C14—H14	119.6
			
C3—N1—O1—C1	176.83 (17)	C7—C8—C9—C14	178.56 (19)
N1—O1—C1—C2	178.10 (15)	C14—C9—C10—C11	−2.1 (3)
O1—C1—C2—C2^i^	176.3 (2)	C8—C9—C10—C11	176.85 (18)
O1—N1—C3—C6	178.15 (15)	C14—C9—C10—C5	178.64 (16)
O1—N1—C3—C4	−2.3 (3)	C8—C9—C10—C5	−2.4 (3)
O2—C5—C6—C7	177.98 (17)	O2—C5—C10—C9	−176.58 (16)
C10—C5—C6—C7	−2.5 (3)	C6—C5—C10—C9	3.8 (3)
O2—C5—C6—C3	−0.4 (3)	O2—C5—C10—C11	4.2 (3)
C10—C5—C6—C3	179.16 (17)	C6—C5—C10—C11	−175.34 (18)
N1—C3—C6—C5	7.8 (3)	C9—C10—C11—C12	0.5 (3)
C4—C3—C6—C5	−171.77 (19)	C5—C10—C11—C12	179.74 (19)
N1—C3—C6—C7	−170.53 (17)	C10—C11—C12—C13	1.5 (4)
C4—C3—C6—C7	9.9 (3)	C11—C12—C13—C14	−2.0 (4)
C5—C6—C7—C8	−0.4 (3)	C12—C13—C14—C9	0.3 (3)
C3—C6—C7—C8	178.04 (18)	C10—C9—C14—C13	1.7 (3)
C6—C7—C8—C9	1.8 (3)	C8—C9—C14—C13	−177.2 (2)
C7—C8—C9—C10	−0.4 (3)		

Symmetry codes: (i) −*x*+1, −*y*+1, −*z*.

### Hydrogen-bond geometry (Å, °)

**Table:**

*D*—H···*A*	*D*—H	H···*A*	*D*···*A*	*D*—H···*A*
O2—H2···N1	0.82	1.84	2.557 (2)	145

## References

[bb1] Atwood, D. A. & Harvey, M. J. (2001). *Chem. Rev.* **101**, 37–52. 10.1021/cr990008v 11712193

[bb2] Dong, W.-K., He, X.-N., Dong, C.-M., Wang, L., Zhong, J.-K., Chen, X. & Yu, T.-Z. (2007). *Z. Kristallogr. New Cryst. Struct.* **222**, 289–290.

[bb3] Dong, W.-K., He, X.-N., Sun, Y.-X., Xu, L. & Guan, Y.-H. (2008). *Acta Cryst.* E**64**, o1917. 10.1107/S1600536808028468 PMC2959278 21201125

[bb4] Okabe, N. & Oya, N. (2000). *Acta Cryst.* C**56**, 1416–1417. 10.1107/s0108270100012786 11118970

[bb5] Sheldrick, G. M. (1996). *SADABS* University of Göttingen, Germany.

[bb6] Sheldrick, G. M. (2008). *Acta Cryst.* A**64**, 112–122. 10.1107/S0108767307043930 18156677

[bb7] Siemens (1996). *SMART* and *SAINT* Siemens Analytical X-ray Instruments Inc., Madison, Wisconsin, USA.

